# Cognitive Dissonance, Personalized Feedback, and Online Gambling Behavior: An Exploratory Study Using Objective Tracking Data and Subjective Self-Report

**DOI:** 10.1007/s11469-017-9808-1

**Published:** 2017-09-20

**Authors:** Michael Auer, Mark D. Griffiths

**Affiliations:** 1 neccton Ltd., Office 404 Albany House, 324 Regent Street, London, W1B 3HH UK; 2 International Gaming Research Unit, Psychology Department, Nottingham Trent University, 50 Shakespeare Street, Nottingham, NG1 4FQ UK

**Keywords:** Behavioral tracking, Gambling, Cognitive dissonance, Gambling expenditure, Online gambling

## Abstract

Providing personalized feedback about the amount of money that gamblers have actually spent may—in some cases—result in cognitive dissonance due to the mismatch between what gamblers actually spent and what they thought they had spent. In the present study, the participant sample (*N* = 11,829) was drawn from a Norwegian population that had played at least one game for money in the past six months on the *Norsk Tipping* online gambling website. Players were told that they could retrieve personalized information about the amount of money they had lost over the previous 6-month period. Out of the 11,829 players, 4045 players accessed information about their personal gambling expenditure and were asked whether they thought the amount they lost was (i) more than expected, (ii) about as much as expected, or (iii) less than expected. It was hypothesized that players who claimed that the amount of money lost gambling was more than they had expected were more likely to experience a state of cognitive dissonance and would attempt to reduce their gambling expenditure more than other players who claimed that the amount of money lost was as much as they expected. The overall results contradicted the hypothesis because players without any cognitive dissonance decreased their gambling expenditure more than players experiencing cognitive dissonance. However, a more detailed analysis of the data supported the hypothesis because specific playing patterns of six different types of gambler using a machine-learning tree algorithm explained the paradoxical overall result.

Gambling is a popular activity found in many cultures (Calado and Griffiths 2016). Surveys have reported that most individuals gamble but do so infrequently (e.g., Bernstein 1996; Wardle et al. 2012), and that most people engage in gambling at some point during in their lives (Meyer et al. 2009; Orford et al. 2003). In Great Britain, nationally representative surveys have found that over two-thirds of the population have engaged in at least one type of gambling in the previous 12 months (Wardle et al. 2012) including both offline and online gambling. A small proportion of gamblers become overly involved in terms of the amount of money and time they spend (e.g., Kessler et al. 2008; Petry et al. 2005). However, internet gambling is accessible 24/7, and potentially negative psychosocial impacts occur among a small minority of individuals. This is because in addition to individuals’ vulnerability factors, various structural and situational characteristics (e.g., accessibility, affordability, and anonymity) may increase the risk of individuals developing a gambling problem (Griffiths 2003; McCormack and Griffiths 2013). Consequently, gamblers need to be educated about how to play more responsibly, and vulnerable groups need to be protected. One way in which to get players to gamble more responsibly is the use of personalized feedback about their actual gambling behavior.

Responsible gambling tools (e.g., limit-setting tools, pop-up messages, personalized feedback, temporary self-exclusions) are a way of facilitating players to gamble in a more responsible manner (Auer and Griffiths 2013; Griffiths et al. 2009; Harris and Griffiths 2017). In two studies using behavioral tracking data, Auer and Griffiths (2014, 2015a) showed that a feedback pop-up message which appeared after 1000 consecutive online slot games had a small but significant effect on the number of players who terminated their current playing session (i.e., significantly more gamblers ceased their playing session after seeing a pop-up message informing them of how many consecutive games on a slot machine they had played compared to controls).

Personalized feedback that informs gamblers about their past playing behavior incorporating a longer time period than just the current session has been empirically investigated in three real-world studies using behavioral tracking data (Auer and Griffiths 2015b, 2016a; Wohl et al. 2017). Auer and Griffiths (2015b) studied the behavior of online gamblers in relation to their voluntary use of a responsible gaming behavioral tracking tool compared with a matched control group of gamblers (that had not used the behavioral tracking tool) on the basis of age, gender, playing duration, and theoretical loss (i.e., the amount of money wagered multiplied by the payout percentage of a specific game played [Auer et al. 2012; Auer and Griffiths 2014]). The results demonstrated that online gamblers receiving personalized feedback spent significantly less money and time gambling in comparison to those that did not receive personalized feedback (i.e., the matched controls).

An experimental study conducted by Auer and Griffiths (2016a) with online gamblers from the Norwegian operator *Norsk Tipping* manipulated the effect of three different types of personalized feedback (personalized feedback, normative feedback, and/or a recommendation). The players were randomly assigned to the specific types of feedback. Compared to the control group (receiving no feedback at all), all groups that received some kind of feedback significantly reduced their gambling behavior as assessed by theoretical loss, amount of money wagered, and gross gaming revenue. The results supported the hypothesis that personalized behavioral feedback enables behavioral change in gambling but that normative feedback did not appear to change behavior significantly more than personalized feedback.

Wohl et al. (2017) investigated the use of a responsible gambling tool that provided personalized feedback to players about how much they had won or lost during a three-month period. Using tracking data from electronic gaming machine (EGM) players provided by the Canadian gambling operator *Ontario Lottery and Gaming*, Wohl et al. found that when players were asked to state whether they thought that the actual amount lost was more or less than they had expected, players who underestimated their losses (i.e., those who lost more money than they thought) reduced the amount they wagered and the amount they lost in the subsequent three months. These results suggest that informing gamblers about their expenditure appears to change subsequent behavior.

Providing information about the amount of money that gamblers have actually spent may—in some cases—result in cognitive dissonance due to the mismatch between what gamblers actually spent and what they thought they had spent. According to the theory of cognitive dissonance (Festinger 1957), individuals tend to seek consistency among their cognitions. Dissonance occurs when there is an inconsistency between attitudes or behaviors. To reduce dissonance, individuals then have to do something to eliminate the dissonance. Festinger (1957) stated that existence of cognitive dissonance (i.e., being psychologically uncomfortable) motivates individuals to try reducing their dissonance in order to achieve consonance. As far as the present authors are aware, no previous study has applied the theory of cognitive dissonance to the perception of gambling behavior.

The present study explored changes in actual gambling behavior by examining whether the amount of money lost gambling was (i) more than expected, (ii) about as much as expected, or (iii) less than expected. For the purposes of the present paper, it was assumed that the answer to this question determined the degree of cognitive dissonance due to the difference between subjective and objective loss of money while gambling (i.e., the greater the difference between what the person thought they had spent versus what they had actually spent would mean greater cognitive dissonance). The methodological approach of the present study was exploratory. However, it was hypothesized that players who claimed that the amount lost was more than they had expected were more likely to experience a state of cognitive dissonance and would therefore attempt to reduce the amount of money they spend gambling compared to players who claimed that the amount of money lost was as much as they expected.

## Methods

### Participants

The participant sample was drawn from the population that had played at least one game for money on the *Norsk Tipping* online platform (*Instaspill*) during April 2015. A total of 11,829 players were randomly selected from 69,631 players that fulfilled the selection criteria (see next section). Of these, 8182 were males (69.1%) and 3647 were females (30.9%). The mean average age was 40.52 years (SD = 13.19 years). Approximately 25% of the customers were younger than 30 years, and 25% were aged over 50 years. There was no significant age difference between males and females (*t* = 1.376, df = 7194, *p* = 0.169). The 11,829 players were sent an email which notified them about the availability of information that was personally relevant to them. It was up to players to click on a link in the email and retrieve the information if they so wished.

### Sampling

The participants only comprised players who had a net loss across all games in the past month before the study commenced (i.e., recent winners were excluded because the goal of the study was to research cognitive dissonance among players who had recently lost). Those who had self-excluded and/or taken a break from gambling were also excluded from subsequent analyses because these players had not gambled. More specifically, the sample was drawn based on the amount lost across all games (online casino, sports betting, lottery, etc.). The amount of money lost by each player was computed by simply subtracting the total amount wagered from the total amount won. The overwhelming majority of players lost only small amounts of money. Therefore, in order to examine the impact of messaging on high-intensity players, there was an oversampling of high-intensity gamblers.

### Personalized Feedback

A simple personalized message was sent to the 11,829 players that said: “How much do you think you spent on gambling recently? Our records show that you lost [XXX] NOK last month.” In addition, players were also presented with an illustrated line chart that contained the monthly values for their personal losses over the previous six-month period. Players were also told that they could retrieve the information any time during the following month. Out of the 11,829 players, 4045 players clicked on the link in the email and viewed their personalized information. After players viewed their personal loss, they were also asked a series of questions. Among them they were asked whether they thought the amount lost was (i) more than expected, (ii) about as much as expected, or (iii) less than expected. As noted above, it was assumed that the response to this question determined the degree of cognitive dissonance (i.e., the greater the difference between subjective and objective loss of money while gambling, the greater the cognitive dissonance).

### Analysis

Assessing whether personalized feedback resulted in the desired behavioral change meant that behavioral change had to be assessed via specific variables and via specific time periods. In the present study, it was decided that gambling behavior seven days prior to the intervention would be compared with gambling behavior seven days after the intervention message was read. This is because changes over a shorter time period would most likely only be due to chance, and changes over longer time periods would not be expected based on the type of feedback provided.

The measure of behavior that was used to assess gambling behavior was theoretical loss (TL). TL is the amount of money wagered and risked (i.e., amount of money staked multiplied by the probability of winning). The TL statistic was computed as “TL_after” minus “TL_before” divided by “TL_before.” This statistic reflects the change in behavior seven days after the message was read as a percentage of the behavior 7 days before the message was read. This procedure helps assess the individual change as independent from the intensity of play as much as possible. A negative value indicates a decrease in gambling behavior and a positive value indicates an increase in gambling behavior. A value of 0 means that no change in gambling behavior occurred at all. A value of (say) − 0.5 means that the gambling behavior decreased by 50% compared to seven days before. The statistic ranges from − 1 to +infinity. A value of − 1 means that the player did not engage in gambling seven days after the message was read. A value of + 10 means that the gambling behavior increased 1000% in the seven days after the message was read compared to seven days before the message was read.

## Results and Preliminary Discussion

In total, 63% of the players reported that the amount lost was about as much as they expected. Three in 10 players (30%) reported that they lost more than expected, and 7% reported that they lost less than expected (see Table 1). Players who reported that they had lost more than expected also spent more money 30 days prior than the other two groups (*X* ^2^ = 80.018, df = 1, *p* < 0.001). The breakdown of differences between gender and age are shown in Tables 2 and 3 respectively.

**Table 1:** Percentages of players (*n* = 4045) who reported they had gambled more or less money than they expected or had spent about the same

	Median theoretical loss	Number
More than expected	774	1236 (31%)
About as much as expected	507	2547 (63%)
Less than expected	377	262 (6%)
Total	556	4045 (100%)

The percentage of females reporting that they had spent more than they expected (see Table 2) was higher than in males (*X* ^2^ = 12.358, df = 2, *p* < 0.002). The median loss for females 30 days before was NOK 696 and the corresponding amount for males was NOK 364 (*X* ^2^ = 207.15, df = 1, *p* < 0.001)

**Table 2:** Percentages of players (*n* = 4045) who reported they had gambled more or less money than they expected or had spent about as much as expected by gender

	More than expected	About as much	Less than expected	Total
Female	403 (34%)	701 (60%)	71 (6%)	1175
Male	833 (29%)	1846 (64%)	191 (7%)	2870
Total	1236 (31%)	2547 (63%)	262 (6%)	4045

The average age across the two first groups was 42 years (see Table 3), and players who reported that they lost less than expected were on average 39 years which was statistically significant (*F* = 6.246, df = 4043, *p* = 0.0125)

**Table 3:** Percentages of players (*n* = 4045) who reported they had gambled more or less money than they expected or had spent about as much as expected by age

	Mean age	Number
More than expected	42	1236
About as much as expected	42	2547
Less than expected	39	262
		4045

Given that the aim of the present study was to explore whether cognitive dissonance leads to change in actual gambling behavior, it was assumed that players who spent more gambling than they thought should decrease the amount of money they spend gambling compared to others. The decrease in the amount of money spent gambling would be the most obvious way for players to reduce cognitive dissonance. If players behave as expected, then those players who reported that the amount of money they had lost was more than expected should decrease the amount of money spent gambling more than players who claim that it is about as much as expected. However, this is not the case. On the contrary, players who reported that the amount of money they had lost was more than they expected actually decreased their gambling expenditure less than those who reported it was about as much as expected (see Table 4). Players who reported that the feedback amount was even less than they expected decreased their gambling behavior the most (see Table 4). These results contradict the hypothesis that cognitive dissonance leads to decreased gambling expenditure (*Χ* ^2^ = 18.105, df = 2, *p* < 0.0001).

**Table 4:** Change in theoretical loss among players (*n* = 4045) seven days after they received personalized feedback about their gambling behavior

	Change in theoretical loss	Number
More than expected	− 35%	1236
About the same	− 44%	2547
Less than expected	− 56%	262
Total	− 42%	4045

However, the change statistic which expresses the change in behavior after the message as a percentage of the behavior before the message was read is not entirely independent of the intensity of play. For this reason, further analysis assessed this difference among groups of equal intensity of play. Consequently, players were divided into two groups: a group of the 90% lowest spenders and a group of the 10% highest spenders. Table 5 shows the median change in theoretical loss separately for the two gambling intensity groups. Players who thought their actual monetary losses were higher than what they expected actually decreased their expenditure compared to those who thought their actual monetary losses were about as high as they expected. This was true among the 90% of lower spenders (*n* = 3635) as well as among the 10% highest spenders (*n* = 410). This was significant for the players with the 90% lowest gambling expenditure (*Χ* ^2^ = 15.586, df = 2, *p* < 0.001) but not significant for the players with the 10% highest gambling expenditure (*Χ* ^2^ = 2.0139, df = 2, *p* < 0.3653).

**Table 5:** Change of theoretical loss seven days after accessing a personalized message among high-intensity gamblers (*n* = 410) and low-intensity gamblers (*n* = 3635)

Intensity group	Answer	Change TL	TL before	Number
Low 90%	More	− 35%	204	1075
	About as much	− 44%	188	2320
	Less	− 56%	167	240
Top 10%	More	− 34%	1891	161
	About as much	− 45%	2254	227
	less	− 65%	1981	22

This means that players who reported that they had lost more money than they expected decreased their gambling expenditure less than players who reported that their monetary losses were about what they expected. In order to better understand this paradox, further data analysis was carried out. More specifically, a recursive tree algorithm was applied. This is a machine-learning method that groups data according to predefined criteria. In the present study, the players’ opinion concerning the actual amount of money lost gambling was used to train the algorithm. The main aim was to determine differences between players who reported that they lost more money than expected and players who reported that they lost about as much as expected. For this reason, the third group of players who reported having lost less than they expected was discarded from further analysis. The goal of the algorithm was to search for groups of players who predominantly chose one of the two possible answers. Six different groups of gamblers with unique patterns of play were extracted using the algorithm. Table 6 describes the pattern (average values) for each of the six groups. The row labeled “Response” contains the dominant answer provided in that particular group. The median seven-day change across all players who answered the question was − 42%. The most significant decrease lay within Group 6, which is also the by far the largest group with 1745 players out of 3783 (46%). This group’s median change was − 69% and therefore contributed the most to the overall median change of − 42%.

**Table 6:** Behavioral segmentation on players (*n* = 3783) using a recursive tree algorithm

	Group 1	Group 2	Group 3	Group 4	Group 5	Group 6	Total
Response	More	More	About as much	About as much	About as much	About as much	
7-day TL change	− 34%	− 36%	− 22%	− 31%	− 9%	− 69%	− 42%
Loss (in NOK)	− 769	− 730	− 1591	− 444	3195	− 280	− 592
Playing days	13	14	17	11	17	5	10
Lottery	29%	31%	27%	51%	14%	55%	43%
Sports betting	19%	1%	14%	7%	24%	5%	9%
VLT	5%	0%	7%	4%	11%	2%	4%
Casino	25%	2%	33%	12%	39%	11%	19%
Scratchcard	13%	59%	5%	12%	4%	20%	16%
Age	32	46	49	44	48	39	42
Gender	26%	42%	20%	31%	10%	35%	29%
Past exclusions	30%	9%	40%	13%	43%	12%	22%
TL trend	51%	31%	29%	25%	23%	27%	28%
*Playscan* rating	62%	100%	100%	0%	84%	22%	48%
*N*	177	162	1051	520	128	1745	3783

*Group 1*: These players predominantly answered that they spent more than they thought and therefore are expected to experience cognitive dissonance. These are young players (mean = 32 years), with above average losses (NOK 792) who prefer sports betting and playing casino games. The response variable labeled “TL trend” indicates whether the theoretical loss over the past six months is steadily increasing. On average, 28% of players show a significant increase. In this group, 51% of players show a significant increase. Players in Group 1 also self-excluded (30%) more often in the past than the average player (22%). The response variable labeled “*Playscan* rating” indicates the percentage of players who were categorized “at risk” of problem gambling by the behavioral tracking system *Playscan* used by the gambling operator

*Group 2*: These players predominantly answered that they spent more than they thought they had and therefore are expected to experience cognitive dissonance. These are female players with above average losses (NOK 730) who prefer gambling on scratchcards. They are also at risk of problematic gambling as identified by the behavioral tracking tool *Playscan* used by the gaming operator

*Group 3*: These players predominantly answered that they spent about as much as they thought and therefore are not expected to experience cognitive dissonance. These are male players with the highest losses (NOK 1591) across all six groups who prefer casino games and have had voluntary self-exclusions from gambling in the past. They are also at risk of problem gambling as identified by *Playscan*

*Group 4*: These players predominantly answered that they spent about as much as they thought and therefore are not expected to experience cognitive dissonance. These are lottery players with low losses (NOK 444)

*Group 5*: These players predominantly answered that they spent about as much as they thought and therefore are not expected to experience cognitive dissonance. These are male players who engage in casino games, sports betting, and VLT gambling. They have had voluntary self-exclusions from gambling in the past. However, they also have had a net win (NOK 3195) over the last week before the message was sent as can be seen by the positive loss value. These players are very specific as they managed to generate a net win over the last week, although their gambling involvement is very high. Their answer can be explained as the feedback, which informs them about high losses over the last months is not in line with their recent experiences during which they managed to win

*Group 6*: These players predominantly answer that they spent about the same as they thought and therefore are not expected to experience cognitive dissonance. These are lottery and scratchcard players with low involvement (NOK 280) and a low number of playing days in the last month (*n* = 5). This is by far the largest group of players in the present study.

## General Discussion

The present study investigated the effect of cognitive dissonance on gambling behavior in a group of real-world players at a real online gambling site. Players were provided with visual and numerical feedback about their gambling losses and then asked if their actual monetary loss was more than they expected, about as much as expected, or less than expected. The present authors hypothesized that players who claimed that the amount lost was more than they had expected were likely to experience a state of cognitive dissonance and would therefore attempt to reduce their gambling expenditure more than other players who claimed that the amount of money lost was as much as they expected. Overall, the results contradicted the hypothesis because players without any cognitive dissonance decreased their gambling expenditure more than players experiencing cognitive dissonance. However, a more detailed analysis of the data using a machine-learning algorithm supported the hypothesis because specific playing patterns of six different gambling groups that were identified explained the paradoxical overall result.

Group 1’s cognitive dissonance is a consequence of their above average losses, the upward trend of the past six months’ theoretical loss, and the fact that they had already self-excluded from gambling in the past. Group 2 had the highest percentage of females and as noted previously, females were more likely to experience cognitive dissonance. Group 3 most likely comprises players who are not entirely honest about their gambling expenditure. They have the highest losses, prefer playing casino games, and have had voluntary self-exclusions in the past. Group 4 comprises lottery players whose gambling involvement is very low. It is therefore understandable that these players are not surprised by the displayed losses and do not experience cognitive dissonance. Group 5 is very similar to Group 3. However, these players recently experienced a net win. Therefore, their recent gambling experience (in the past seven days) was very different from the feedback they received. Their answer is therefore in line with the data.

Group 6 is similar to Group 4. However, these gamblers play infrequently. Due to the low gambling involvement, it is likely that these players’ gambling expenditure decreases from one week to the next. The decrease in behavior (reflected by a 69% drop in theoretical loss) is not so much connected to the personalized information or the answer given, but to their playing habit. It could easily be the case that that these players did not gamble in the week after the personalized message was sent as they only gamble five days a month on average. Apart from Group 6 and Group 4 (who both comprise infrequent lottery players) the most significant decrease in gambling behavior occurred in Group 1 (34%) and Group 2 (36%). These are the two groups that are likely to have experienced cognitive dissonance.

Wohl et al. (2017) published the only comparable study to the present study with participants who played electronic gaming machines (EGMs). Players who underestimated their losses (i.e., those who lost more money than they thought) reduced the amount they wagered and the amount they lost in the subsequent three months. After performing a detailed machine-learning analysis, the original hypothesis that players who experience cognitive dissonance decrease their gambling expenditure more significantly than players who do not experience cognitive dissonance was supported by the data. The overall counterintuitive pattern is more easily explained when examining the six subgroups of players identified by the learning tree algorithm. The reasons for the overall paradoxical pattern are likely to be a (i) highly involved group of casino players who do not appear to experience cognitive dissonance, (ii) highly involved group of casino players who recently experienced a net win which is not in line with the feedback which informs them about a loss, and (iii) large group of lottery players who decrease their gambling, a consequence of their infrequent playing behavior.

In previous studies, personalized feedback has been shown to change player behavior (Auer and Griffiths 2015a; Auer and Griffiths 2015b; Auer and Griffiths 2016a; Griffiths et al. 2009; Wood and Wohl 2015). Several studies have also shown that players’ subjective loss estimation is significantly biased (Wohl et al. 2017; Auer and Griffiths 2016b) which further underlines the importance of objective information. The present study utilized the theory of cognitive dissonance to explain behavioral change as a consequence personalized feedback.

Compared to laboratory studies, the sample size was considerably large and is one of the few studies to compare objective and subjective data from the same gamblers. However, there are of course limitations. The players only comprised *Norsk Tipping* customers who were sampled at one point in time and were all Norwegian. Although players were asked about their perceived losses, the results are still inferential rather than definitive. Additionally, behavioral change was only determined over the period of one week. For that reason, it is difficult to conclude whether the results can be applied to other areas or platforms. There are a variety of possible avenues for further research. Future empirical studies should use the underlying methodology outlined in the present study and be applied to other gambling operators across different countries and jurisdictions. Gambling behavior should be also assessed over longer periods of time, and the objective information collected using tracking data should be complemented with self-recollected information about players’ cognitive beliefs and motivations to play. Unlike many other studies in the area of gambling research, the present study was carried out in a real-world setting with real players in real time and is one of the very few studies that have directly compared objective and subjective data from the same gamblers. Given these strengths, the present study makes a novel and innovative addition to the gambling studies literature.
